# Epileptic Seizure from Ginkgo Nut Intoxication in an Adult

**DOI:** 10.1155/2020/5072954

**Published:** 2020-01-28

**Authors:** Yoshinori Kosaki, Hiromichi Naito, Tsuyoshi Nojima, Atsunori Nakao

**Affiliations:** Department of Emergency, Critical Care and Disaster Medicine, Okayama University Graduate School of Medicine, Dentistry and Pharmaceutical Sciences, Okayama, Japan

## Abstract

The ginkgo tree is a well-known, highly adaptable urban plant. Ginkgo nuts are the product of the ginkgo tree. Interior ginkgo nuts are cooked and served in Asian countries; however, the potential toxicity of the gingko nuts is not commonly known. Herein, we report a 48-year-old male patient experiencing acute convulsions presumably due to overconsumption of gingko nuts. The patient was transferred to our department after several episodes of acute generalized tonicclonic seizures lasting approximately 30 seconds each and starting one hour before the visit. The patient also complained of vomiting, vertigo, diarrhea, and tremors in both upper limbs following the seizures. Elevated 4-O-methylpyridoxine (312 ng/mL), low blood pyridoxal phosphate (2.4 *μ*g/L), and low vitamin B1 (20 ng/mL) levels were found in the blood analysis. No other remarkable abnormalities were detected. We diagnosed the patient with ginkgo nut intoxication, and he was orally administered 400 mg of pyridoxal phosphate. His symptoms resolved after treatment, and no seizures recurred thereafter. Our report may help raise awareness of the clinical presentation and management of this intoxication among emergency physicians.

## 1. Introduction

The ginkgo tree is a well-known, highly adaptable urban plant with gorgeous, golden, fan-shaped leaves. Ginkgo nuts are the product of the ginkgo tree. In Japan, ginkgo nuts are cooked until the shells split, and the interior nut, which is a luminescent green color, is roasted, salted, and eaten at celebrations, often served alongside sake and beer. People also take ginkgo seeds as herbal medication due to their expectorant and antitussive functions. However, the potential toxicity of the gingko seed is not as commonly known. As herbal medicine and oriental cuisine become more popular worldwide, emergency physicians should know about the potential dangers of overconsumption of traditional oriental food and how to promptly treat problems resulting from gingko seed consumption. Although the mechanism underlying the convulsion-inducing effect of gingko nuts is clearly understood, 4-methoxypyridoxine contained in the gingko seed can also contribute to its harmful toxicity [1].

Previously published ginkgo seed poisoning cases have usually involved children exhibiting repetitive, potentially fatal seizures [2–5]. Herein, we report a 48-year-old male patient experiencing acute convulsions presumably due to overconsumption of gingko nuts. Our report may help raise awareness of the clinical presentation and management of this intoxication among emergency physicians.

## 2. Case Presentation

A 48-year-old man was transferred to our department after several episodes of acute generalized tonicclonic seizures lasting approximately 30 seconds each and starting one hour before the visit. The patient also complained of vomiting, vertigo, diarrhea, and tremors in both upper limbs following the seizures. His past medical history was unremarkable, with no previous episodes of convulsion. He was not taking any medications. However, the patient's wife reported that he has been an alcoholic for three months and his nutritional status was poor.

At the time of presentation, he was oriented with coherent speech and without paralysis. His vital signs were pulse rate 74 beats/min, blood pressure 142/76 mmHg, respiratory rate 18 breaths/min, body temperature 37.1°C, and oxygen saturation 98%. Neurological examination, blood gas analysis, toxicological screening, and cerebrospinal fluid examination results were unremarkable. His laboratory data were unremarkable, including normal serum ammonia level (34 *μ*g/dL), but his lactate level was mildly elevated (2.5 mmol/L). Electrocardiogram and radiographs were examined to rule out pulmonary and cardiovascular causes and were found to be normal. No ascites was seen. Suspecting encephalophagy related to alcoholism, magnetic resonance imaging was performed; however, no pathological changes were noticed. Electroencephalography examination showed no slow and sharp waves or spikes.

After obtaining his detailed medical history, we determined that the patient had consumed approximately 80 fried ginkgo seeds in appetizers approximately six hours before the seizure occurred. Analysis of the patient's stored serum revealed an elevated concentration of 4-O-methylpyridoxine (312 ng/mL), low blood pyridoxal phosphate (2.4 *μ*g/L), and low vitamin B1(20 ng/mL) levels. We diagnosed the patient with ginkgo nut intoxication, and he was orally administered 400 mg of pyridoxal phosphate. His symptoms resolved after treatment, and no seizures recurred thereafter.

## 3. Discussion

In our patient, ginkgo seeds containing the poisonous ingredient 4-O-methylpyridoxine, also called ginkgotoxin, could have triggered vomiting and epileptic convulsions associated with vitamin B6 (pyridoxal phosphate) deficiency. 4-O-Methylpyridoxine is considered an anti-vitamin B6 compound because it is related structurally to vitamin B6 and interferes with its metabolism, function, and biosynthesis [5]. Vitamin 6 is a coenzyme of glutamate decarboxylase, but 4-O-methoxypyridoxine competitively inhibits the formation of the suppressive neurotransmitter *γ*-aminobutyric acid (GABA) and is synthesized from glutamate by the enzyme glutamate decarboxylase. Therefore, in the presence of vitamin B6 deficiency, 4-O-methoxypyridoxine may be responsible for epilepsy because the central nervous system (CNS) neurons become abnormally irritable due to decreased GABA production [6]. As 4-O-methoxypyridoxine is repeatedly excreted via the enterohepatic circulation, ginkgo nut intoxication-induced convulsions may become repetitive [5].

Our patient purchased commercial gingko nuts in shells obtained in Japanese grocery store and consumed after fried. As 4-O-methoxypyridoxine is stable and does not inactivate with heat, prepared gingko nuts with heat are still considered to be poisonous.

A previous literature review indicated that gingko nut intoxication is especially common in children who accidentally ingest them. Seven to 150 pieces for children and 40 to 300 pieces for adults are the ranges for overdose. Poisoning time ranges from one to 12 hours after consumption. Symptoms and signs mainly focus on the CNS and include vomiting, coma, drowsiness, fear, convulsions, lags in response, increased temperature, difficulty breathing, dark-purple complexion, contracted or dilated pupils, slowness in reaction to light, stomachache, diarrhea, and rising counts of white blood cells and neutrophilic granulocytes. Generally, the cause and the degree of poisoning are closely associated with age, physique, and intake quantity.

Our case showed a decreased pyridoxal phosphate level and increased 4-O-methoxypyridoxine in the blood six hours after ginkgo nut overconsumption. In our patient, besides deficiency of other vitamins, alcohol overconsumption may have changed the release and/or absorption of neurotransmitters in the brain. In addition, alcohol binds to the brain's GABA receptors, leading to a relaxed feeling as the neurons fire less quickly. Although the mechanisms of gingko nut intoxication-induced seizure have not fully been clarified, 4-O-methoxypyridoxine is most likely responsible for epilepsy due to the imbalance between GABA and glutamate [7]. Emergency physicians must become knowledgeable regarding the clinical features of gingko nut toxicity to avoid further unnecessary examination, cost, and time expenditures.

## References

[1] Miwa H., Iijima M., Tanaka S., Mizuno Y. (2003). Generalized convulsions after consuming a large amount of gingko nuts. *Epilepsia*.

[2] Jang H.-S., Roh S. Y., Jeong E. H., Kim B.-S., Sunwoo M. K. (2015). Ginkgotoxin induced seizure caused by vitamin B6 deficiency. *Journal of Epilepsy Research*.

[3] Kajiyama Y., Fujii K., Takeuchi H., Manabe Y. (2002). Ginkgo seed poisoning. *Pediatrics*.

[4] Hasegawa S., Oda Y., Ichiyama T., Hori Y., Furukawa S. (2006). Ginkgo nut intoxication in a 2-year-old male. *Pediatric Neurology*.

[5] Wada K., Ishigaki S., Ueda K., Sakata M., Haga M. (1985). An antivitamin B6, 4′-methoxypyridoxine, from the seed of Ginkgo biloba L.. *Chemical & Pharmaceutical Bulletin*.

[6] Kästner U., Hallmen C., Wiese M., Leistner E., Drewke C. (2007). The human pyridoxal kinase, a plausible target for ginkgotoxin from Ginkgo biloba. *The FEBS Journal*.

[7] Kobayashi D., Yoshimura T., Johno A., Ishikawa M., Sasaki K., Wada K. (2015). Decrease in pyridoxal-5′-phosphate concentration and increase in pyridoxal concentration in rat plasma by 4′-O-methylpyridoxine administration. *Nutrition Research*.

